# Duration of Cattle Ranching Affects Dung Beetle Diversity and Secondary Seed Removal in Tropical Dry Forest Landscapes

**DOI:** 10.3390/insects15100749

**Published:** 2024-09-27

**Authors:** Juan J. Morales-Trejo, Wesley Dáttilo, Gustavo Zurita, Lucrecia Arellano

**Affiliations:** 1 Instituto de Ecología, A.C. (INECOL), Red de Ecoetología, Carretera Antigua a Coatepec 351, El Haya, Xalapa 91073, Ver., Mexico; coanocyte@gmail.com (J.J.M.-T.); wesley.dattilo@inecol.mx (W.D.); 2 División de Posgrado en Ciencias, Instituto de Ecología, A.C., Km 2.5 Camino Antiguo a Coatepec 351, Xalapa 91073, Ver., Mexico; 3 Instituto de Biología Subtropical (IBS), Universidad Nacional de Misiones (UNaM)—CONICET, Av. Tres Fronteras 183, Puerto Iguazú 3370, Misiones, Argentina; gazurita@conicet.gov.ar; 4 Facultad de Ciencias Forestales, Universidad Nacional de Misiones, Bertoni 128, Eldorado 4405, Misiones, Argentina

**Keywords:** diversity, abundance, disturbance gradient, species turnover, ecological processes, seeds

## Abstract

**Simple Summary:**

The use of inadequate cattle ranching practices (e.g., fire, agrochemical applications, long term extensive grazing, lack of paddock rotation in pastures) could have negative consequences on biodiversity and ecological functions. In this study, the influence of cattle ranching duration on the diversity of dung beetles and seed removal was evaluated in pastures having different times of establishment in a tropical dry forest landscape. Dung beetle species richness was similar along the gradient of grazing ages, but the diversity of common and dominant species declined with increasing grazing age. Seed removal was mainly carried out by an exotic species. Although native dung beetles persisted at low abundances along this gradient, the consequences of land use changes and inadequate practices in similar landscapes could lead to their disappearance.

**Abstract:**

Cattle ranching is an economic activity responsible for the loss of large extensions of tropical dry forest around the world. Several studies have demonstrated that the use of inadequate practices of this activity in tropical forests (e.g., fire, agrochemicals, and lack of rotational grazing systems of cattle in pastures) have negative consequences on dung beetle diversity and their ecological functions. In the present study, the influence of the cattle ranching duration gradient on the diversity of dung beetles and seed removal was evaluated. This study was carried out in pastures with different times of establishment of cattle ranching (between 4 and 40 years) in a tropical dry forest of Mexico. Overall, the species richness of dung beetles was similar along the gradient of grazing ages. However, the diversity of common (q1) and dominant (q2) species decreased and was associated with an increasing abundance of exotic species and a decreasing abundance of native species. Seed removal was mainly carried out by four beetle species, among which the exotic species *Digitonthophagus gazella* was the most important. The results establish that the duration of cattle ranching primarily influences the composition of dung beetle communities, as reflected in changes in the structure and function of their assemblages in the pastures. Although native dung beetles persist at low abundances along this gradient, the consequences of land use changes are undeniable in other similar ecosystems where these species could definitively disappear.

## 1. Introduction

Tropical dry forests are of ecological and conservation interest because of their high biodiversity and level of endemism [1]. However, at the same time, the environmental conditions (e.g., climate, vegetation, and soil type) are considered excellent for the establishment of crops such as exotic grasses and maize used to feed cattle [2,3]. The productive activities and land uses associated with cattle ranching have caused a considerable reduction in tropical dry forest cover, with less than 10% of its original extent remaining in Mexico [4,5,6,7].

Among the effects of land use changes related to conventional or extensive livestock grazing are the loss and/or reduction of species diversity and changes in the species composition of different taxa (i.e., soil macrofauna [8,9,10,11,12]). Macrofauna play an important role in the maintenance of soil health [13], including dung beetles (Coleoptera: Scarabaeinae), which use vertebrate dung, small cadavers, fruits, mushrooms, and detritus to feed and nest [14]. Through these activities, dung beetles remove and bury organic matter, favoring the bioturbation, fertilization, aeration, and regeneration of soil, in addition to controlling cattle flies and contributing to secondary seed dispersal [15]. However, dung beetles are very sensitive to ecosystem disturbances caused by human activities [16,17,18]. Therefore, any alterations to the microclimate conditions or the resources of a site can affect the ecological functions, and the ecosystem services associated with these functions because of changes to species abundance, diversity, or composition [19,20].

The secondary dispersal of seeds defecated by vertebrates is one of the most important ecological processes for the regeneration of tropical forests. This process is mainly carried out by mice, ants, and dung beetles. In the case of dung beetles, this occurs randomly when the portions of excrement removed by dung beetles contain some seeds inside [21,22,23]. Seed removal by dung beetles is differentially associated with the size of the individuals and the size of the seeds. Seeds are dung contaminants from the beetle’s perspective, and larger beetles relocate larger portions of dung. Thus, secondary seed dispersal by dung beetles is negatively related to seed size and positively related to beetle size [24]. Another factor is the type of dung relocation [14,22,24,25,26,27]. For example, paracoprid species (tunnelers) bury dung under or to the side of their food source. They are generally diverse, abundant, and large and can thus relocate large amounts of dung. In addition, they are less selective of the dung that they process and, as a result, exclude few seeds [22,24,28,29,30,31]. Telecoprid species (rollers) cut pieces of dung, make balls with them, and move them specific distances before buying. The horizontal distribution of seeds by these beetles can reduce the mortality and density-dependent competition of seeds in the primary site of deposition [22,24,31,32]. Additionally, the germination probability of some seeds is favored by their transport to better microsites (underground or far from desiccation) [23,24] by beetles. Finally, endocoprid species feed and nest in dung [14,33], are generally small, and have no role as secondary seed dispersers.

Cattle ranching is an important agent of land use change that influences local environmental conditions that beetles depend on through modifying the structure and dynamics of ecosystems [34,35,36]. Previous studies showed that when cattle ranching activities intensified or occurred over an extended period of time, the food resources available for dung beetles changed the structure (both richness and composition) of dung beetle communities [37,38,39]. In the Neotropics, the scarcity of grassland-adapted species is a characteristic; however, exotic species such as *D. gazella* have occupied empty niches in these environments and have been successful in their colonization of these environments. Additionally, the spread of cattle ranching areas and the corresponding increase in sunny areas throughout the tropical dry forest, along with the growing abundance of exotic resources, can favor the existence of dung beetle species shared between forests and pastures [40]. The situation is different in other regions, such as the Palearctic, where cattle have occupied open spaces for a long time and there are species adapted to this condition and manure.

Although the appearance and continuous supply of an exotic resource (i.e., cow manure) in pastures would imply a scenario in which native beetles obtain an additional food resource [41,42], native dung beetle species involved in secondary seed dispersal may sometimes disappear as a consequence of the introduction of livestock [38] or, if they remain, may be reduced in number [43]. Therefore, the process of secondary seed dispersal by these organisms could be negatively affected in human use landscapes.

Conventional and extensive cattle ranching has generated an increase in pasture areas that could also promote the arrival of exotic dung beetle species and their colonization of vacant niches, especially in the coastal plains of Mexico [44,45,46,47]. Exotic species are more efficient in the use of cow manure than native species [48] and are better adapted to open sites [18,40,49]. Thus, the temporal prolongation of cattle ranching practices could result in the eventual replacement of native species by exotic species that, at the same time, could replace the manure removal function [50,51] and secondary seed dispersal carried out by native species [37,52]. The topic of secondary seed dispersal by dung beetles has not been addressed from the perspective of the duration of cattle ranching in tropical dry forest landscapes. For this reason and given that dung beetles are a good model for applied biodiversity studies [53,54] and ecosystem functions [15,55,56], these insects were selected as a model for the present study.

In this study, we explored the influence of the duration of cattle ranching (between 4 and 40 years) on the structure of dung beetle assemblages (i.e., abundance, richness, and diversity qD) and secondary seed dispersal in a tropical dry forest of Mexico. We also explored changes in the abundance of exotic dung beetle species and seed removal according to relocation strategy. Our biological hypothesis was that cattle ranching activities, as an agent of land use change, influence local conditions for dung beetle assemblages because they modify the ecosystem (structure and dynamics) [57]. Therefore, when cattle ranching is intensified or is practiced over a prolonged time period, the availability of food resources for dung beetles, changes, and the diversity and composition of species also change [37]. The appearance and continuous supply of an exotic resource (cow manure) in the pastures would represent a scenario in which native species could find an additional food resource [41,42]. However, this scenario could also favor the arrival of exotic species of dung beetles that are more efficient in using cow manure [48].

Based on this hypothesis, (1) we expected the abundance, richness, and diversity of dung beetles to decrease with the duration of cattle ranching, (2) the abundance of exotic species to increase and to provide the opposite pattern for native species along the gradient of duration of cattle ranching. In addition, (3) we expected exotic species to have a more relevant role in seed removal than native species with increased duration of cattle ranching.

## 2. Materials and Methods

### 2.1. Study Area

Sampling was performed in five ejidos in the municipality of La Huerta located in the northern area of influence of the Chamela-Cuixmala Biosphere Reserve (CCBR) in the state of Jalisco, Mexico (19°23′/19°38′ N and −104°57′/−105°5′ W). An ejido is an extension of communal land that is usually located on the outskirts of a town where agricultural, livestock, or other activities are carried out, and was originally provided by the government to benefit the community. The region has an average elevation of 145 masl. Its climate type is Aw0i (warm sub-humid), according to the modified Köppen classification [58,59], with an average annual temperature of 25 °C [59]. This climate type is the driest of all the sub-humid climate types, with an annual average rainfall of 750 mm. The rainy season extends from June to October [60]. The dominant vegetation is tropical dry forest [61]. However, due to human activity over the last 50 years, most primary vegetation has been converted to croplands and pastures. Thirty percent of the area in the municipality is dedicated to agricultural activities [62]. The transformation of the natural vegetation has left behind remnants of native vegetation with different levels of conservation immersed in an anthropogenic matrix.

In the region of Chamela, Jalisco, Mexico, 69 species of mammals have been observed in the fragments of the tropical dry forest. Among the most important and that could contribute to maintaining abundant populations of dung beetles are the white-tailed deer (*Odocoileus virginianus sinaloae*), collared peccary (*Pecari tajacu sonoriensis*), coyote (*Canis latrans vigilis*), ocelot (*Leopardus pardalis nelsoni*), cougar (*Puma concolor aztecus*), ringtail (*Bassariscus astutus consitus*), and cottontail (*Sylvilagus cunicularius insolitus*) (see [63]).

### 2.2. Selection of Study Plots

**Figure 1:**
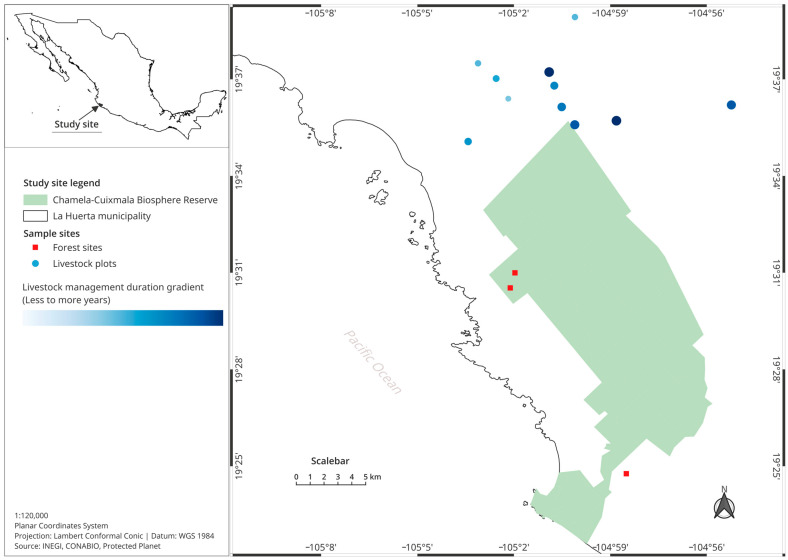
Location of the study plots in the buffer zone of the Chamela-Cuixmala Biosphere Reserve. Color gradation and increasing size of the circles represent the increasing duration of cattle ranching in each pasture.

Given the landscape heterogeneity of the dry tropics, one-hectare plots were carefully researched (10,000 m^2^) and selected to maximize their homogeneity with respect to land use (sites with regular cattle ranching), soil type (shallow and rocky regosols), and topographic position (i.e., an average south-facing slope) [3]. With the aid of landowners, 11 plots with between 4 and 40 years of cattle ranching activity were selected. In addition, three plots of conserved forest (plots without cattle ranching use) also were selected. The shortest distance between the closest pair of sites was 1.4 km (Figure 1).

### 2.3. Sampling of Dung Beetles

Sampling was performed between July and August 2012 (rainy season) during the months with the highest abundance of dung beetles, according to samplings previously performed by the authors. In each plot, six baited pitfall traps were distributed along two transects separated by 50 m [64] and buried at soil level. In each trap, 60 g of fresh cattle manure without remains of deworming treatments from the same locality was used. The trap design is described in [53] (Figure S1A). Our traps have an unknown attraction radius that may be species-specific, meaning that the communities inhabiting the regional matrix may influence the collections from each site, to varying extents.

After 48 h, the traps were inspected, and trapped specimens were taken to the laboratory for cleaning and identification. Individuals were identified by Leonardo Delgado and Fernando Escobar (Instituto de Ecología A.C. [INECOL], Mexico). The reference collections were deposited in the Chamela-UNAM Tropical Biological Station (Estación de Biología Tropical de Chamela-UNAM) and the Eco-Ethology Department (Red de Ecoetología) at INECOL.

### 2.4. Seed Removal

During the rainy season of 2012, six plots measuring 19 cm in height and 32 cm in diameter were buried in each plot 50 m from each other. They were cut longitudinally, allowing them to be separated into two parts, but were joined with adhesive tape (to recover their original form) before burial. Pots were filled with 9 kg of soil from the same ranch to imitate the soil used by beetles as a nesting substrate. On top of the soil, a plastic grid with an opening of 2.7 × 2.4 cm was placed, and 1.5 kg of fresh cattle manure was placed on the grid. The pots were then fitted with an inclined fiberglass roof to avoid saturating the soil with rainwater (taken from [65], Figure S1B). In each dung pat, 40 brown-colored plastic beads of different sizes were placed (referred to herein as “seeds” according to the method of [21]). Natural seeds were not used since this would require excluding other organisms that also remove seeds from dung, such as mice and ants [21,66]. In contrast, plastic beads are not removed by these animals and their size, shape, and the number of seeds used can be controlled. Plastic beads can also be reused [24].

The size range and number of seeds used in this experiment were based on data from a previous experiment (L. Arellano, pers. comm.) in which seeds naturally deposited by cattle were counted and measured in a sample of 10 dung pats per plot. We included seeds of different sizes that represented at least 1.5% of the total seeds (and sizes) found in the analyzed dung pats, which ranged from five to nine millimeters (Figure S1C). To define the total number of seeds in the dung pats, we estimated the percentage that each seed size represented in our experiment: one 5 mm seed, ten 6 mm seeds, nineteen 7 mm seeds, nine 8 mm seeds, and one 9 mm seed.

To quantify horizontal dispersal and to complement the prior experiment on seed removal by dung beetles, two additional experiments were placed in each plot. The first consisted of placing fresh dung pats (each weighing 100 g) containing six seeds at 50 m from each pot. The second experiment consisted of situating six fresh dung balls (each weighing 5 g) with one 5 mm seed at a distance of 5 m from the 100 g dung pats and from each other, forming a circle around the dung pats (Figure S1C). This was done because rollers are more selective when they construct balls and avoid incorporating unpalatable or large seeds in their balls [22,27]. Based on [21], red 50 cm threads were attached to seeds to help locate them.

All experiments had a duration of 72 h in the field, after which they were reviewed. To inspect the pots, the roofing was removed, and each dung pat (1.5 kg) was placed in a tray. The beetles and seeds inside were counted, and the beetle species were identified. Then, each pot was extracted from the soil and separated into halves by removing the adhesive tape. Each half was carefully inspected, removing the soil with a spatula to search for beetles and locate seeds. When beetles and/or seeds were found inside, they were extracted and placed in recipient trays to identify and count them [65].

### 2.5. Data Analysis

Data from the forests (n = 3) were included in the calculation of sampling coverage, in the evaluation of the beetles’ assemblage structure, and in the calculation of the different orders of diversity. Since these sites had not experienced cattle ranching, data from these sites were only used as reference points.

#### 2.5.1. Sample Completeness

The sample completeness in each plot was estimated using the sample coverage estimator proposed by [67]. This estimator is based on the proportion of the total beetle assemblage represented by the captured species. A value close to 100% indicates that the sample is complete according to the capture method (baited traps) and sampling effort. The calculation of sample coverage was carried out using the iNEXT package (version 2.0.12) [68,69] in R software (version 4.4.1) [70].

#### 2.5.2. Beetle Diversity

Species diversity in each plot was estimated using the qD approximation of [71], which considers species abundance. Specifically, it measures the diversity that a community would have if composed of *i* equally common species. The orders of diversity corresponding with species richness (zero-order diversity: q0), first-order diversity (Shannon’s exponential: q1), and second-order diversity (inverse Simpson’s index: q2) were also calculated. First-order diversity indicates species evenness when species are weighted proportionally to their exact abundance in the assemblage, while second-order diversity describes the common species in the assemblage [72]. The diversity measures were calculated in the iNEXT package (version 2.0.12) in R software [67,69].

#### 2.5.3. Beetle Assemblage Structure

The non-metric multidimensional scaling (NMDS) technique was used to evaluate and compare dung beetle composition among plots using the Bray–Curtis dissimilarity index. This type of ordination analysis is one of the most used and often summarizes more information on fewer axes than other direct ordination techniques, enabling the results to be more easily interpreted [73]. The analysis was carried out using the metaMDS function in the vegan package (version 2.4–6) [74,75] in R software [69].

#### 2.5.4. Relationship of Dung Beetle Abundance and Diversity with Cattle Ranching Duration

To evaluate whether the abundance and diversity of dung beetle species (q0, q1, and q2) were related to cattle ranching duration, simple linear regressions and exponential regressions were performed based on the proposals of [76,77,78]. Using this analysis, the relative abundance of species or group of species (exotic-native) was used as the dependent variable and cattle ranching duration as the independent variable.

The models with the *p*-values closest to 0 (*p* < 0.05) and highest coefficient of determination (R^2^) were selected. This latter coefficient is also used to discriminate between significant models and select those with the best fit.

#### 2.5.5. Seed Removal by Dung Beetles and Cattle Ranching Duration

To evaluate seed removal by dung beetles associated with cattle ranching duration, we estimated the percentage of seeds (with respect to the total of n = 270/plot) moved out of the dung pats in the pot. Experiments and two complementary experiments were used for estimations in addition to those seeds found on the soil or buried (modified from [43]). The calculated percentage was used as a response variable and the effect of grazing as an independent variable in a simple linear regression.

## 3. Results

### 3.1. Dung Beetle Diversity

In total, 4285 dung beetles belonging to 11 species and seven genera were collected (Table 1). In the pastures, 3566 individuals belonging to 11 species (nine native and two exotic) were collected, and 719 individuals belonging to seven native species in the forests were collected. The exotic species *Euoniticellus intermedius* (Reiche, 1849) and *Digitonthophagus gazella* (Fabricius, 1787) were only recorded in pastures. The most abundant species was *E. intermedius* (51%, 2200 recorded individuals). The highest abundance of paracoprid species was found in forests and in the pastures with the longest duration of cattle use (Table 1). Of the collected species, *Canthon indigaceus* (Harold, 1868) was the only telecoprid species, and it was most abundant in the pastures with the longest duration of cattle use. The sampling coverage per plot was 99%, indicating that the sampling effort was suitable for diversity comparisons (Table 1).

**Figure 2:**
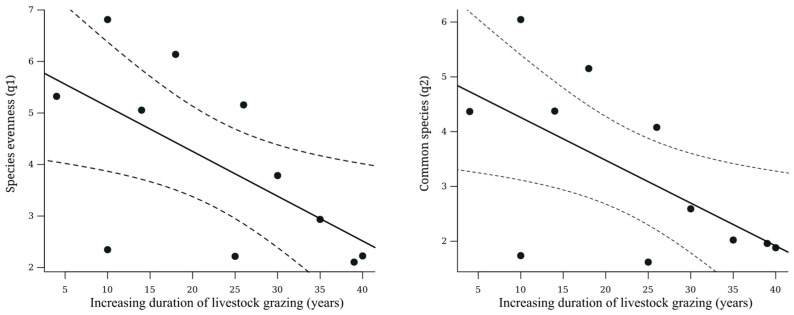
Relationship of species evenness (**left** figure) and common species (**right** figure) with the duration of cattle ranching. The solid line represents the trend of the data and the dashed lines represent the 95% confidence interval.

Both species richness (linear adjustment: R^2^ = 0.08, *p* = 0.39; exponential adjustment: R^2^ = 0.08, *p* = 0.7) and total abundance of individuals (linear adjustment: R^2^ = 0.09, *p* = 0.36; exponential adjustment: R^2^ = 0.18, *p* = 0.46) showed no relation to the gradient of cattle ranching duration. However, the diversity of common (q1) and dominant (q2) species decreased (R^2^ = −0.39, *p* = 0.04 and R^2^ = −0.38, *p* = 0.04, respectively) as the duration of cattle ranching increased (Figure 2 and Table 2).

**Figure 3:**
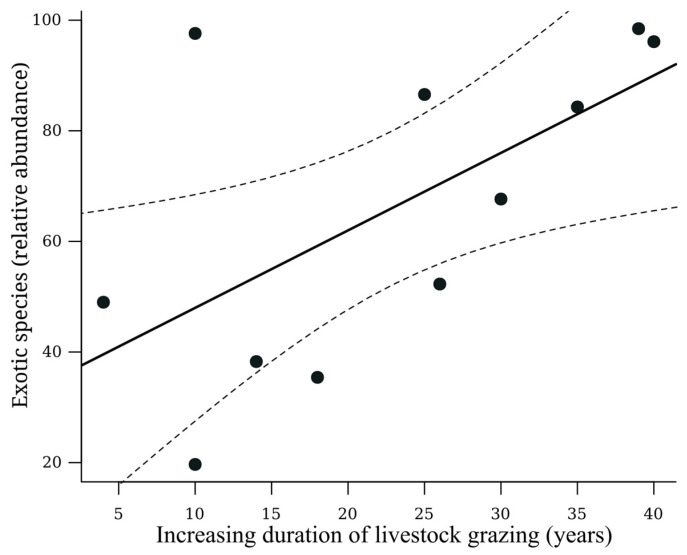
Relationship between the relative abundance of exotic species and the duration of cattle ranching. Fit of the linear regression: R^2^ = 0.38, *p* = 0.04. The solid line represents the trend of the data and the dashed lines represent the 95% confidence interval.

On the other hand, the relative abundance of exotic dung beetle species increased with the duration of cattle ranching (R^2^ = 0.38, *p* = 0.04) (Figure 3).

**Figure 4:**
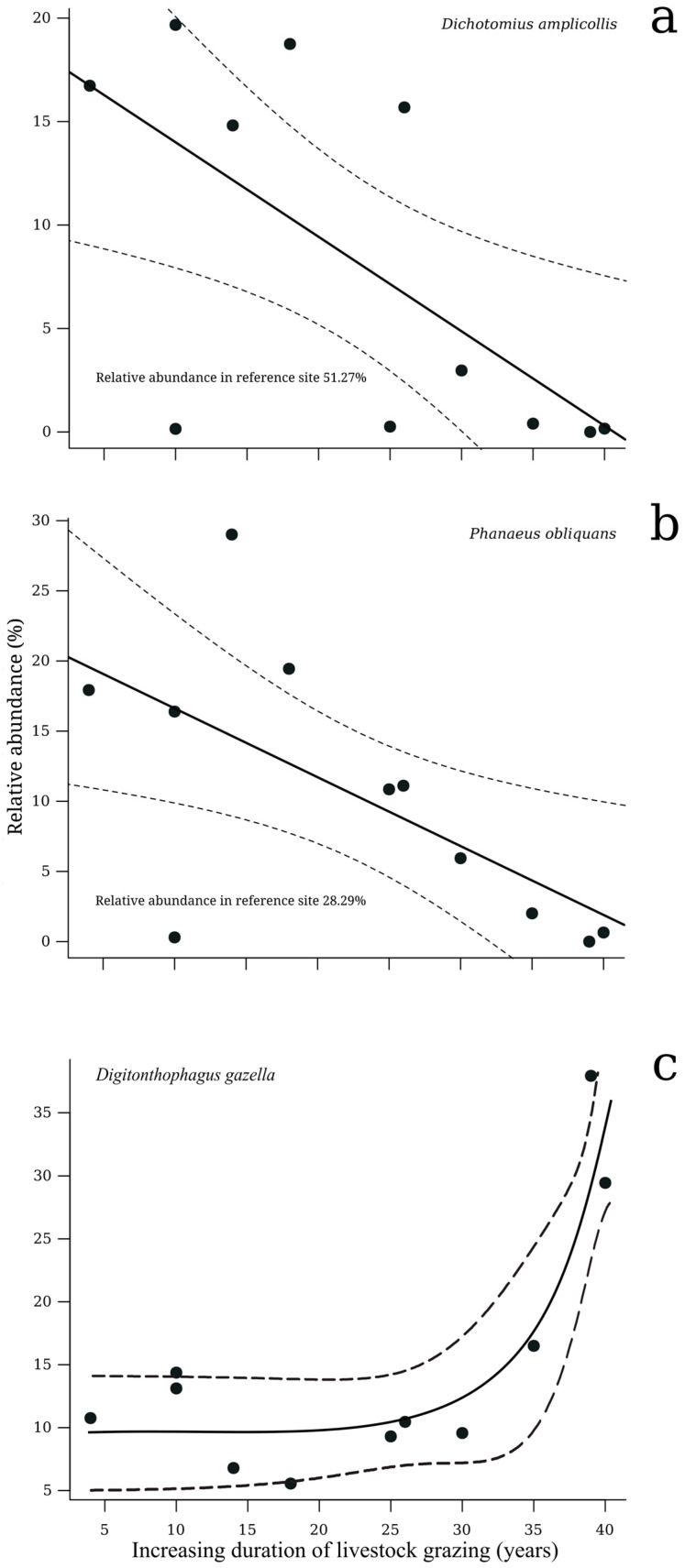
Relationship of the relative abundance of (**a**) *Dichotomius amplicollis* (native species), (**b**) *Phanaeus obliquans* (native species), and (**c**) *Digitonthopagus gazella* (exotic species) with the duration of cattle ranching. The solid line represents the trend of the data and the dashed lines represent the 95% confidence interval.

Only the relative abundance of three species (*Dichotomius amplicollis* (Harold), 1869; *Phanaeus obliquans* Bates, 1887; and *D. gazella*) was significantly related to the duration of cattle ranching. The relative abundance of the native species *D. amplicollis* and *P. obliquans* declined with cattle ranching duration (R^2^ = −0.43, *p* = 0.03 and R^2^ = −0.41, *p* = 0.03, respectively) (Figure 4a,b), whereas the abundance of the exotic species *D. gazella* showed the opposite pattern, in particular after 30 years of land use as pasture (R^2^ = 0.83, *p* = 0.0008) (Figure 4c).

**Figure 5:**
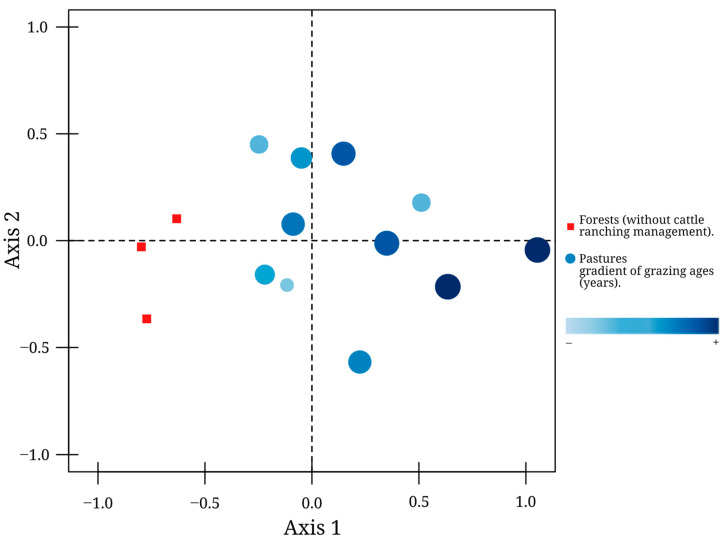
Grouping of plots according to their species composition. The gradient of color and the size of the circles represent the increasing duration of cattle ranching in each site.

When comparing dung beetle composition, the plots clearly formed two groups according to species composition: forests and pastures (NMDS, stress = 0.11). In the case of pastures, the plots formed a close group along a visible gradient of lower to higher duration of cattle ranching duration (axis 1) (Figure 5).

### 3.2. Relationship between Seed Removal and Cattle Ranching Duration

**Figure 6:**
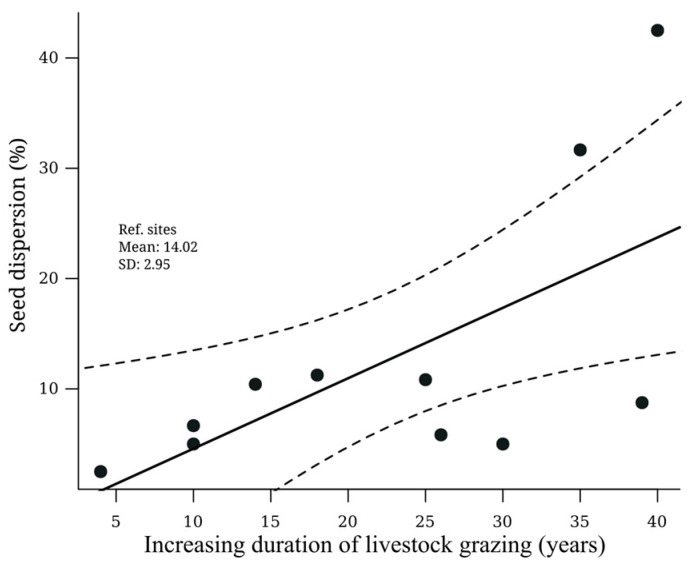
Relationship of the percentage of seed dispersal with the duration of cattle ranching. Average and standard deviation of the percentage of seed dispersal in forests as compared to reference values are shown. The solid line represents the trend of the data and the dashed lines represent the 95% confidence interval.

Only four beetle species were associated with seed removal along the gradient of cattle ranching duration: the native species *Dichotomius colonicus* (Linnaeus, 1767), *P. obliquans*, and *C. indigaceus,* and the exotic species *D. gazella* (Figure 6).

Pastures with more years of cattle ranching activities showed a percentage of seed removal up to 17 times higher than the others (R^2^ = 0.40, *p* = 0.036). Of the 2970 seeds placed in pastures, only 337 (11%) were removed: *C. indigaceus* (the only roller) removed a total of 135 seeds, and the paracoprids (*D. colonicus, D. gazella*, and *P. obliquans*) removed 202 seeds. The percentage of seeds removed increased with the duration of cattle ranching; pastures with four years of cattle ranching had a percentage of seed removal 2.6 times lower than the pasture with 10 years of cattle ranching activities. Likewise, the pasture with 14 years of cattle ranching activities had a percentage of seeds removed 4.1 times greater than the pasture with four years of cattle ranching.

## 4. Discussion

Many studies show that forest replacement has negative consequences on dung beetle diversity [79,80,81,82,83,84,85,86,87,88,89,90]. Thus, we expected a decline in dung beetle diversity with the increasing duration of cattle ranching activities. However, no effect of the duration of cattle ranching on species richness was evidenced.

Regarding species diversity, the decrease in dung beetle diversity (q1, q2) over the gradient of cattle ranching duration is likely a consequence of an increase in the dominance of a few exotic species. Native forest species are replaced by colonizing species better adapted to the conditions of open sites [39,48,91,92,93]. Increased cattle ranching activities impart an important change in species composition and abundance, as was observed.

The negative influence of cattle ranching activities on species composition and abundance observed in this study was reflected in the relation between exotic/native species and the cattle ranching gradient; although the most abundant species (exotic) in pastures were not found in the studied forests, their abundance increased with increasing cattle ranching activity, while the abundance of native species declined. In addition, there was an unexpected presence and abundance of *D. colonicus* in cow dung inside of the Chamela-Cuixmala Biosphere Reserve (CCBR) forests and inside of other forests situated in CCBR adjacent localities. This species is typical of open areas and disturbed sites. Perhaps its presence was related to the sampling of forests, because CCBR is under the influence of cattle ranching activities. Yet, it is adjacent to El Zarco (a major cattle ranch) and to several ejidos such as San Mateo and Ranchitos, where some of the pasture plots were located, and where the stream “Colorado” was historically located as cattle crossing area. Additionally, the establishment of the Chamela-Cuixmala Biosphere Reserve (13,142 ha in 1993) is a relatively recent event.

Native species are functionally important and contribute to the removal of organic matter in forests and other land use areas, and their conservation is not in question. However, exotic species that tolerate microclimatic conditions of pastures could become functionally important in livestock landscapes due to their high abundance [37]. Our study shows that, in pastures, the exotic beetles *D. gazella* and *E. intermedius* are the most abundant species along the gradient of the duration of cattle ranching. The abundance of exotic beetles is determined by their capacity to adjust to the dominant environmental conditions of pastures, which are open sites with abundant cattle manure [48,51,93]. This finding is also explained by the relaxation of inter-specific competition with native species, since none were observed where they shared similar daily activity, size, and relocation strategies (same ecological niche); rather, they seemed to be occupying empty niches in the cattle pastures [45], although their abundances in the dung pats may represent up to 100% of the individuals found there, which could negatively affect beetle populations.

Interestingly, the introduction of the exotic species *D. gazella* and *E. intermedius* to the American continent dates to the 1970s, when they were used to help remove manure and control cattle flies in pastures in southern Texas, United States [44]. Since then, because of their reproductive and dispersion capacity, these exotic species have colonized a large part of Mexico, moving through pastures along the coasts [44,45]. In any case, the presence of cattle manure favors the dispersion, establishment, and persistence of both native and exotic dung beetle species, which both contribute to and perform different ecosystem functions [37,94], enhancing grassland soil fertility [95] and successful seedling emergence [96].

In our study, the native species associated with seed removal were two large paracoprids, *Dichotomius colonicus* (19–28 mm) and *P. obliquans* (10–21 mm), that have little selectivity for the dung that they process and, in consequence, exclude few seeds when burying dung [22,28,29,30,31]. They are considered efficient in dung burial because of their size [26,97,98,99,100,101], which also favors their seed removal capacity [26,41,49,97]. The maximum seed size dispersed by these beetles is close to their body length [102], and all seeds used in this study were smaller than the body size of these species.

Native paracoprid dung beetles were most abundant in forests and also in the oldest pastures (with a greater supply of manure). Despite having lower abundances than the exotic species (paracoprids, small species), they still play an important ecological role because of their biomass and dung burial capacity.

In the pastures with the longest duration of cattle use, *C. indigaceus*, the only native telecoprid species, was abundant and had an important role in seed removal. This species is likely favored by the large areas without trees in these pastures and because of its preference for cattle manure. However, the increase in seed removal by dung beetles in pastures may also be related to the abundance of exotic species, such as *D. gazella,* which was clearly the dominant species in the plots with more than 30 years of cattle ranching use. This exotic species is smaller (approx. 10–13 mm) than the native species (e.g., *D. amplicollis*, *D. colonicus*, *P. obliquans*, and *Phanaeus tridens*, >20 mm approx.). However, its small body size could be compensated for by its high abundance in the plots [41], enabling it to actively participate in the removal of manure and, subsequently, in the process of seed removal [21,26].

Notably, we found a low rate of seed removal in pastures (<13% in total) compared to previous studies carried out in tropical forests and with frugivorous animals (>44%) [24,26,29,43,103,104,105,106,107,108,109,110,111]. These results could be explained by the lower abundance of dung beetle species available to carry out this function in the studied pastures, the low seed rain in these environments with respect to tropical forests, or the mismatch between the season of greatest seed consumption by cows, who are the primary dispersers at these sites (dry season) and the presence of dung beetles (wet season), which are in diapause during the dry months (Arellano, personal communication). Furthermore, in the sites where cattle ranching was practiced for a long time or was intensive, the abandoned pastures are dominated by herbaceous plants and weeds that delay or detain succession [112,113].

Previous studies on secondary seed dispersal by dung beetles in pastures are scarce and were carried out in the temperate zone of Belgium using horse dung [32] and in the Western Palaearctic (at 17 study sites in 10 countries and 11 biogeographic zones) [114,115]. In the first article, the beetles that removed seeds were mainly endocoprid species (genus *Aphodius* spp.), and the removed seeds were small and corresponded with grasses (*Poa* spp. and *Agrostis* spp.). The study mainly focused on the success of seed germination following the intervention of dung beetles. In the second study, data from a large-scale, multi-site experiment were described and the visualization of data for each country and locality is complex. A third study was carried out in the temperate Atlantic biogeographical region in two nature reserves using cattle, horse, and sheep dung and *Galium aparine, Alopecurus myosuroides*, and *Poa pratensis* seeds. Therefore, there are no previous studies of seed removal in tropical pastures and/or with cattle as the primary seed dispersers with which we can compare our results.

Additionally, seed removal carried out by dung beetles is a process of vital importance because it may result in a reduction of the spatial clustering of plants and increased seedling establishment by reducing the risks of predation and mortality, directing the dispersal to more favorable locations for germination, decreasing the scramble competition for space and nutrients by seedlings [15,22,43,64,104,116], and potentially contributing to the regeneration of tropical dry forests [94]. This is mainly because cattle not only feed on pasture grasses but also on woody forest species (mainly Fabaceae such as *Vachellia pennatula* and *Guazuma ulmifolia*, which are dispersed by cattle). These seeds are ingested and subsequently deposited along with the manure used by dung beetles [117,118]. Therefore, if the seeds are dispersed far from the source or buried at a depth of several centimeters (along with the manure) by beetles [119], as was observed to occur in this study, the possibility of germination is higher because of their arrival to sites with a lower probability of predation [15]. It has been observed, for example, that the seeds of *Pithecellobium dulce* (guamúchil) are predated by ants along the studied gradient.

In addition, it is important to emphasize that the patterns found herein correspond to a dry forest landscape wherein seven large native species and genera of beetles persist along a gradient of cattle ranching disturbances lasting up to 40 years. These species are efficient in the burial of dung [49,99,120], including *Copris lugubris* Boheman, 1868, *D. amplicollis*, and *P. obliquans*. However, the exotic species appear to be more successful. Although this could be explained by the better adaptation of exotic species to open sites and the presence of resources such as cow manure, it should be noted that the persistence of native species might also be due to the influence of the proximity of the Chamela-Cuixmala Biosphere Reserve. The supply of plant propagules enables the diversity of some native dung beetle species associated with forest sites to be maintained [18,40]. In this regard, forests often represent a source of propagules near pastures that, on average, have a tree cover of 50% and in ejidos, such as Los Ranchitos, where forest management is as important an activity as cattle ranching [121], which enables the connectivity of the landscape to be maintained. In addition, as mentioned above, the forest areas are strongly influenced by the nearby cattle ranching activity. For example, the areas near Arroyo Colorado in the Tropical Biological Station were once used as a cattle passage for herding cattle toward Nacastillo.

## 5. Conclusions

Our study shows that the total abundance and species richness of dung beetles were not affected by the duration of cattle ranching. However, the evenness of the assemblage (q1) and common species index (q2) decreased because of an increasing abundance of exotic species. One novel finding is that the process of seed removal was carried out by both exotic and native dung beetles in pastures. Although the present research represents an instantaneous snapshot of the study site at a particular point in time, the tendency toward the slow replacement of native dung beetle species suggests that a change in the actual cattle ranching practices in dry tropical forest landscapes is necessary to mitigate the deterioration and to contribute to the regeneration of this ecosystem.

## Figures and Tables

**Table 1:** Abundance of dung beetle species collected from areas influenced by the Chamela-Cuixmala Biosphere Reserve, Mexico. Duration of livestock grazing in each plot (F1–F3 and P1–P11) is indicated in parentheses.

Species	O	F1 (0)	F2 (0)	F3 (0)	P1 (4)	P2 (10)	P3 (10)	P4 (14)	P5 (18)	P6 (25)	P7 (26)	P8 (30)	P9 (35)	P10 (39)	P11 (40)	T
*Ateuchus rodriguezi* (Borre, 1886)	N	2	0	0	0	1	3	0	7	2	1	5	0	0	0	21
*Canthon indigaceus* Le Conte 1859	N	0	0	0	0	2	2	0	2	0	1	2	22	1	0	32
*Copris lugubris* Boheman, 1868	N	5	3	4	8	30	0	7	23	0	9	56	4	0	6	155
*Dichotomius amplicollis* (Harold, 1869)	N	139	131	100	42	24	1	24	27	1	24	9	2	0	1	525
*Dichotomius colonicus* (Say, 1835)	N	40	33	51	23	15	8	21	3	2	21	7	35	3	13	275
*Digitonthophagus gazella* (Fabricius, 1787)	E	0	0	0	27	16	96	11	8	36	16	29	82	99	182	542
*Euoniticellus intermedius* (Reiche)	E	0	0	0	96	8	556	51	43	299	64	176	337	158	412	1788
*Onthophagus igualensis* Bates, 1887	N	0	0	1	0	0	0	0	0	2	0	0	0	0	0	3
*Onthophagus landolti* Harold, 1880	N	0	0	0	10	0	0	1	3	3	0	0	4	0	0	21
*Phanaeus obliquans* Bates, 1887	N	58	91	56	45	20	2	47	28	42	17	18	10	0	4	438
*Phanaeus tridens* Castelnau, 1840	N	3	2	0	0	6	0	0	0	0	0	1	1	0	0	13
Abundance		247	260	212	251	122	668	162	144	387	153	303	497	261	618	
Zero-order diversity (species richness: q0)		6	5	5	7	9	7	7	9	8	8	9	9	4	6	
First-order diversity (q1)		3.09	2.89	3.15	5.32	6.81	2.34	5.05	6.13	2.21	5.15	3.78	2.93	2.1	2.22	
Second-order diversity (q2)		2.5	2.54	2.85	4.36	6.04	1.73	4.37	5.15	1.61	4.07	2.59	2.02	1.95	1.88	

O = origin of species, N = native, E = exotic; F1–F3 = forest plots; P1–P11 = pasture plots; T = total per species.

**Table 2:** Results of the linear and exponential models evaluating the effect of the duration of livestock grazing on different variables of the dung beetle assemblages. Each line shows the variables analyzed in the function of livestock grazing (excluding those mentioned in the text).

	Linear			Exponential			
Variable ~~Duration of Livestock Grazing	R^2^	*p*-Value		R^2^	*p*-Value		Relationship
Species richness (q0)	0.08	0.39		0.08	0.70		-
Shannon diversity (q1)	0.39	0.04	*	0.39	0.14		Negative
Simpson diversity (q2)	0.38	0.04	*	0.38	0.15		Negative
Total abundance	0.09	0.36		0.18	0.46		-
Exotic species (+)	0.38	0.04	*	0.44	0.10		Positive
*A. rodriguezi* (+)	0.02	0.69		0.00	1.00		-
*C. indigaceus* (+)	0.05	0.52		0.00	1.00		-
*C. lugubris* (+)	0.08	0.39		0.08	0.71		-
***D. amplicollis* (+)**	0.43	0.03	*	0.43	0.11		Positive
*D. colonicus* (+)	0.17	0.22		0.17	0.49		-
***D. gazella* (+)**	0.41	0.03	*	0.83	0.01	**	Positive
*E. intermedius* (+)	0.22	0.14		0.23	0.36		-
*O. igualensis* (+)	0.00	0.87		0.00	1.00		-
*O. landolti* (+)	0.26	0.11		0.00	1.00		-
***P. obliquans* (+)**	0.41	0.03	*	0.41	0.12		Positive
*P. tridens* (+)	0.10	0.34		0.10	0.65		-

* Significant models; ** model selected from two significant results; + relative abundances. Native species *D. amplicollis* and *P. obliquans* and exotic species *D. gazella* are highlighted in bold.

## Data Availability

The data presented in this study are available on request from the corresponding author due to we are using some information of this data base for other publications.

## Supplementary Materials

The following supporting information can be downloaded at https://www.mdpi.com/article/10.3390/insects15100749/s1: Figure S1: A. Design of baited pitfall traps and disposal in field. B. Sampling units to measure ecological functions. C. Disposal in field of seed removal experiments.
