# Supplementary figures and images for: Duration of Cattle Ranching Affects Dung Beetle Diversity and Secondary Seed Removal in Tropical Dry Forest Landscapes
Source: Insects. 2024 Sep 27;15(10):749. doi: 10.3390/insects15100749 (PMC11508350; doi:10.3390/insects15100749)

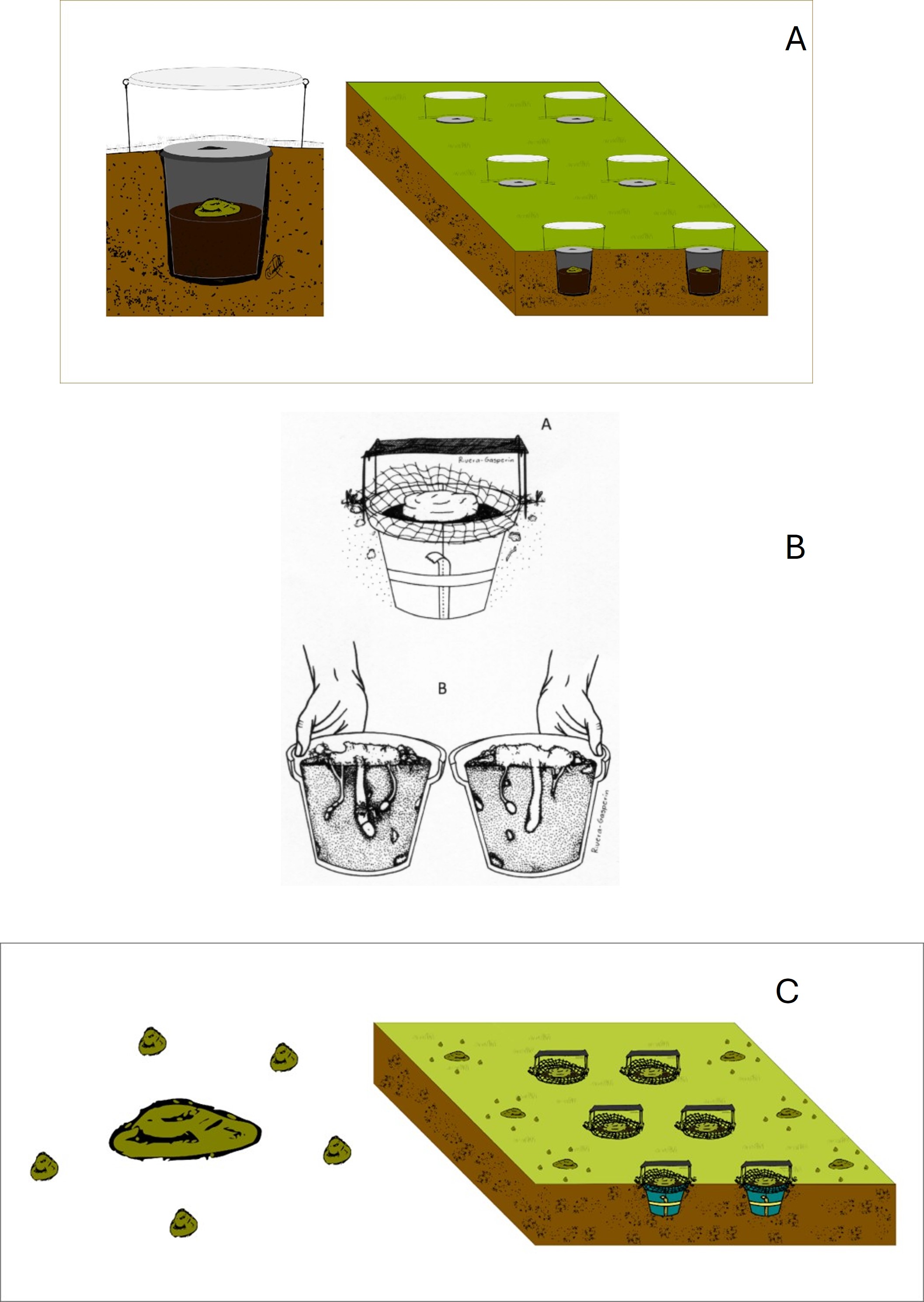

Supplement: Supplementary file 1 [file insects-15-00749-s001.zip › insects-3206193-supplementary.jpg]
